# Immobilization of *Providencia stuartii* Cells in Pumice Stone and Its Application for *N-*Acetylglucosamine Production

**DOI:** 10.17113/ftb.60.01.22.6994

**Published:** 2022-03

**Authors:** Yuniwaty Halim, Devianita Devianita, Hardoko Hardoko, Ratna Handayani, Lucia C. Soedirga

**Affiliations:** 1 Food Technology Department, Universitas Pelita Harapan, Jl. M.H. Thamrin Boulevard, Lippo Karawaci, Tangerang 15811, Indonesia; 2 Faculty of Fisheries and Marine Sciences, Brawijaya University, Jl. Veteran No. 1, Malang 65113, Indonesia

**Keywords:** cell immobilization, chitin degradation, *N-*acetylglucosamine production, *Providencia stuartii*, pumice stone, repeated fermentation

## Abstract

**Research background:**

Shrimp shells contain chitin that can be further processed into *N-*acetylglucosamine, which has been extensively used to treat joint damage. *Providencia stuartii* has a strong chitinolytic activity and may be utilized in the form of immobilized cells in repeated fermentation. Pumice is a porous and rigid stone that offers superior mechanical strength, making it suitable for immobilization.

**Experimental approach:**

In the research submerged fermentation with different pumice stone sizes and pumice stone/growth medium ratios (*m*/*V*) was carried out for 4 days at 37 °C and pH=7.0. The optimum pumice stone size and pumice stone/growth medium ratio (*m*/*V*) were used to determine the optimum fermentation cycle for the production of *N-*acetylglucosamine using immobilized *P. stuartii*.

**Results and conclusions:**

Pumice stones of 1.0 cm×1.0 cm×1.0 cm and pumice stone/growth medium ratio of 1:5 were found to be the optimum conditions for successful immobilization of (90.0±1.6) % cells and production of (331.4±7.3) g/L *N-*acetylglucosamine. The highest *N-*acetylglucosamine concentration of (323.0±2.5) g/L was obtained in the first fermentation cycle, which then decreased and remained stable throughout the last three cycles.

**Novelty and scientific contribution:**

*P. stuartii*, a strong chitinolytic bacterium previously isolated from rotten shrimp shells, was used for the first time in immobilized form to produce *N-*acetylglucosamine. The findings in this research showed the potential use of *P. stuartii* cells immobilized in pumice stone for continuous production of *N-*acetylglucosamine in repeated fermentation.

## INTRODUCTION

Shrimp shells comprise 30-40% protein, 30-50% calcium carbonate and 20-30% chitin, depending on the type of the shrimp (*1*). The monomeric unit of chitin includes *N-*acetylglucosamine, an amino sugar that plays a role in stimulating joint functions and forming the structure of cartilage (*2*). The lack of glucosamine might lead to the symptoms of osteoarthritis, which is often developed in 90% of people above 40 years old (*3*). *N-*acetylglucosamine can be produced through chemical synthesis, enzymatic process or microbial fermentation method (*2*). The chemical synthesis is not necessarily preferred due to its lower yield and environmental issues because strong acids are used (*4*), while enzymatic process poses a great challenge with its high cost of enzyme purification, lower yield and enzyme stability issues (*2*). Hence, the microbial fermentation method is more preferred to produce *N-*acetylglucosamine (*5*).

The microorganisms which can be used to synthesise *N-*acetylglucosamine are those that can produce chitinolytic enzyme to break down chitin into glucosamine (*6*). Previous research has successfully isolated 17 microorganisms that possess chitinolytic activity from rotten tiger shrimp shells. About 8 of them possessed strong chitinolytic activity; however, *Providencia stuartii* was the strongest chitinolytic bacterium isolated (*7*). Chitinolytic index of *P. stuartii* in the previous research was about 4.46 after incubation for 48 h at 37 °C (7) and it is higher than of other chitinolytic bacteria isolated from similar sources (solid and liquid waste of shrimp shells), *i.e. Acinetobacter johnsonii* (chitinolytic index of 2.069) and *Bacillus amyloliquefaciens* (chitinolytic index of 2.084) (*8*). Although *P. stuartii* is pathogenic, a mild heat treatment is sufficient to inactivate it because of its mesophilic nature (*9*). Therefore, *P. stuartii* was considered as a potential producer of high amounts of *N-*acetylglucosamine from shrimp shells.

Cell immobilization is a technique of fixing the cells onto a solid support system, into a solid support matrix or retaining them by a membrane for stability, thus enabling their repeated or continued utilization (*10*). Immobilization also results in a high concentration of cells. Bacterial cells can be immobilized on a solid, porous matrix by entrapment method, which is relatively rapid and simple yet offers high stability of cells (*11*). Immobilization of *P. stuartii* allows the cells to be repeatedly used for the *N-*acetylglucosamine production.

Pumice is a porous and rigid stone with high mechanical strength. The high porosity of about 90% makes it preferable for use in immobilization as it may lead to a large surface area with low commercial cost (*11*). Moreover, pumice, which is primarily composed of silica, is also an ideal immobilization matrix. Its inertness and stability make it reusable (*12*). It was also reported in a previous research that the productivity of immobilized cells in pumice stones is twofold higher than the suspended cell system (*13*).

Pumice stone has been used to immobilize several microorganisms, such as *S. cerevisiae* (*14*), *Penicillium digitatum* (*15*), *Clostridium beijerinckii* NRRL B-593 (*16*) and *Aspergillus niger* (*17*). Furthermore, pumice stones which have been used to immobilize microorganisms can be used for the production of protease (*18*), fructooligosaccharides (*19*), lactic acid (*20*), and for improving β-glucanase productivity (*21*).

In this research, *P. stuartii* cells were immobilized by entrapment method using pumice stone for further use in *N-*acetylglucosamine production from shrimp shells. The objectives of this research are to determine the optimum size of pumice stone, optimum ratio of pumice stone and growth medium (*m*/*V*), as well as the optimum fermentation cycle of immobilized cells used in the fermentation-based production of *N-*acetylglucosamine.

## MATERIALS AND METHODS

### Materials

The main materials used in this research were the shells of whiteleg shrimp (*Penaeus vannamei*) obtained from PT First Marine Seafood, Muara Baru, North Jakarta, Indonesia, the culture of *Providencia stuartii* obtained from previous research (*7*) and the pumice stones from Aquadratic Aquarium, Bandung, Indonesia, as the immobilization matrix. The chemicals and media used in this research were the distilled water, standard *N-*acetylglucosamine (Sigma-Aldrich, Merck, St. Louis, MO, USA), nutrient agar, nutrient broth, bovine serum albumin, Coomassie Brilliant Blue G-250, dipotassium phosphate, potassium dihydrogen phosphate, ammonium sulphate, magnesium sulphate heptahydrate, ninhydrin and pH=7 buffer (all from Merck, Darmstadt, Germany).

### Shrimp shell powder preparation

Shrimp shells were separated from the leftover meat, washed and sun*-*dried for two days. The dried shrimp shells were then crushed into powder using a mill (FCT-Z500; Fomac, Jakarta, Indonesia) and sieved through a 60-mesh sieve, yielding a smooth shrimp shell powder (*22*).

### Immobilization of P. stuartii cells using pumice stone

The immobilization of *P. stuartii* cells began by preparation of pumice stone as the immobilization support. The pumice stones were cut into different sizes of 1.0 cm×1.0 cm×1.0 cm, 1.5 cm×1.5 cm×1.5 cm, and 2.0 cm×2.0 cm×2.0 cm, boiled for 10 min, washed three times, and dried overnight at 60 °C using an oven (UNE 800; Memmert, Schwabach, Germany). The stones were then sterilized using autoclave (Hiclave HVE 50; Hirayama, Saitama, Japan) at 121 °C for 15 min before use. Meanwhile, 1 mL of *P. stuartii* culture was inoculated into 300-mL growth medium. The growth medium used in this research consisted of 2.4 g nutrient broth, 0.09 g KH_2_PO_4_, 0.21 g K_2_HPO_4_, 0.03 g MgSO_4_·7H_2_O, and 2.1 g (NH_4_)_2_SO_4_ in 300 mL distilled water (*23*).

The pretreated pumice was then submerged in the growth medium with pumice stone/growth medium ratio (*m*/*V*) 1:5, 1:10 and 1:15 and left there for 2 h at 37 °C using an incubator (BE600; Memmert). The number of immobilized cells was counted by subtracting the number of unimmobilized cells from the initial number of cells using haemocytometer (*24*).

Submerged fermentation was done for 4 days at 37 °C and pH=7.0 with manual periodic shaking. These values of temperature and pH were reported to be the optimum conditions for the growth of *P. stuartii* (*25*, *26*). Fermentation was carried out by putting the immobilized cells from different treatments into the fermentation medium, consisting of 30 g shrimp shell powder, 0.09 g KH_2_PO_4_, 0.21 g K_2_HPO_4_, 0.03 g MgSO_4_·7H_2_O, and 2.1 g (NH_4_)_2_SO_4_ in 300 mL distilled water (*23*).

To stop the fermentation, the medium containing immobilized cells was then heated at 70 °C for 45 min in a water bath (WNB-14; Memmert), followed by centrifugation at 2800×*g* for 15 min using a centrifuge (MPW e-223; Westertimke, Germany) and filtration through Whatmann No. 1 filter paper. The obtained filtrate was then analysed for its *N-*acetylglucosamine content (*27*). Optimum size of pumice stone and optimum pumice stone/growth medium ratio (*m*/*V*) were then determined based on the *N-*acetylglucosamine concentration obtained from the fermentation.

### Determination of optimum fermentation cycle

The optimum size of pumice stone and optimum pumice stone/growth medium ratio were then used to determine the optimum fermentation cycles. The fermentation was repeated up to four cycles (*28*). Each fermentation cycle was done at 37 °C and pH=7.0 for 4 days, shaken periodically. After each cycle, the fermentation was terminated by heating and the concentration of *N-*acetylglucosamine was quantified using a UV-Vis spectrophotometer (Thermo Scientific™ Genesys™ 10s; Thermo Fisher Scientific, Waltham, MA, USA) at 324 nm (*27*).

### Scanning electron microscopy analysis on immobilization support

Pumice stones with ratio to medium of 1:5 and size of 1.0 cm×1.0 cm×1.0 cm with the *P. stuartii* immobilized cells were collected from the growth medium and dried overnight in the incubator (BE600; Memmert) at 37 °C. The prepared pumice samples were then sent to PT. Qantaz Warna Kreasi, West Java, Indonesia, for the scanning electron microscopy (SEM) analysis. The immobilized pumice stone was observed with a Thermo Scientific™ Quanta™ FEG 650 scanning electron microscope (Thermo Fisher Scientific).

### Data analysis

To determine the optimum fermentation cycle, we used a completely randomized factorial design with five replications. The data were analyzed statistically with Analysis of Variance (ANOVA) using SPSS Software, v. 22.0 (*29*). Further analysis was done using Duncan’s *post hoc* test.

## RESULTS AND DISCUSSION

Cell immobilization is a technique of fixing cells into a support to keep their stability, allowing the possibility of repeated or continued use (*10*). Therefore, we counted the immobilized *Providencia stuartii* cells to make sure that a sufficient amount of them were immobilized into the pumice stones. Table 1 shows the percentage of immobilized *P. stuartii* cells from different treatments. The initial number of *P. stuarti* cells prior to immobilization was 10^7^ CFU/mL, because for fermentation process the required bacterial cell count is about 10^6^-10^7^ CFU/mL (*30*).

**Table 1:** Percentage of *Providencia stuartii* cells immobilized using different pumice stone size and pumice stone/growth medium ratio

Pumice stone size/cm	*m*(pumice stone)/*V*(growth medium)	Immobilized cells/%
1.0×1.0×1.0 1.5×1.5×1.5 2.0×2.0×2.0	1:5 1:10 1:15 1:5 1:10 1:15 1:5 1:10 1:15	90.0±1.6 85.2±3.2 80.8±2.5 81.1±4.6 80.5±1.1 71.6±2.4 79.6±1.1 71.3±1.8 63.0±3.6

Data are presented as mean value±S.D, *N*=27

Table 1 shows that pumice stones can be effectively used as a matrix for cell immobilization with the highest immobilized cell percentage of (90.0±1.6) %. This outcome indicates that most of the bacterial cells had been entrapped in the pumice stone pores. Larger size of pumice stone tends to lead to lower percentage of immobilized cells. This result correlates with previous research on immobilization of *Teredinobacter turnirae* cells for protease production, which stated that pumice stones of smaller size and rougher surface offer superior microenvironmental conditions for cell immobilization (*18*), such as larger contact area and more favourable binding sites for the cell surface structures to interact (*31*), leading to higher percentage of immobilized cells. In addition, a carrier with a large surface area to volume ratio may result in an efficient immobilization, as the cells should first attach to the surface of the support before being progressively entrapped in the pores (*32*).

This result is better than in another research that immobilized *Pseudomonas putida* using pumice particles, where immobilization efficiency was 67.83% (*33*). It is also higher than other research using alginate beads to immobilize chitosan, *i.e.* about 74% (*34*), calcium agar beads and agar beads to immobilize α-amylase, *i.e.* 80 and 63.83%, respectively (*35*). Therefore, it can also be inferred that the macroporous pumice has greater loading capacity than the natural gels.

Higher ratio of pumice stone to growth medium (*m*/*V*) contributes to higher percentage of immobilized cells because it provides larger surface for the immobilization regardless of the types of material (*36*). In this research, pumice stone/growth medium ratio (*m*/*V*) 1:5 offered the most suitable amount of carriers compared to the other treatments.

**Fig. 1:**
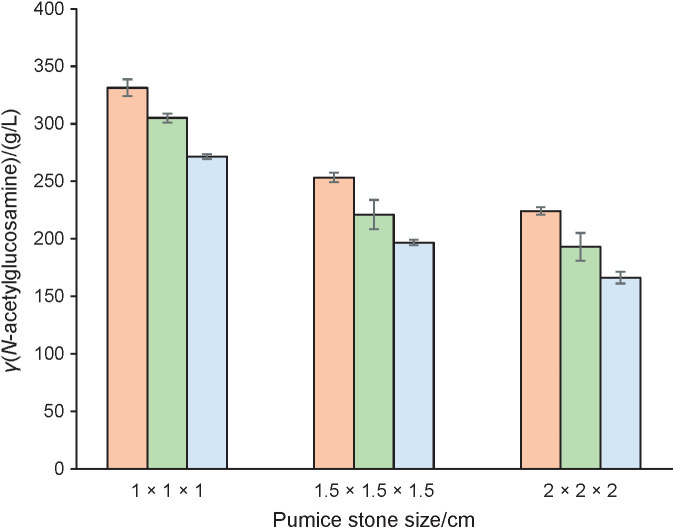
Effect of pumice stone size and *m*(pumice stone)/*V*(growth medium)=1:5 (orange), 1:10 (green) and 1:15 (blue) on *N-*acetylglucosamine concentration obtained after fermentation. Data are presented as mean value±S.D, *N*=27

To know the efficiency of pumice stone in immobilizing *P. stuartii* cells, *N-*acetylglucosamine produced after fermentation was also measured. Fermentation was conducted for 4 days at 37 °C with pH=7.0 of the medium. This temperature and pH were required for optimum growth of *P. stuartii* (*25*, *26*). The results (Fig. 1) show that different pumice stone sizes and different pumice stone/growth medium ratios (*m*/*V*) affect the production of *N-*acetylglucosamine.

Fig. 1 shows that the production of *N-*acetylglucosamine decreases with an increase of pumice size. The highest *N-*acetylglucosamine concentration was obtained from the cells immobilized in the pumice stones of 1.0 cm×1.0 cm×1.0 cm, which contained the highest percentage of immobilized cells. The porous structure and a large surface area of the supporting material promote an efficient immobilization, resulting in high yield of products (*37*).

On the contrary, another research found that protease production first increases before it starts to decrease as the pumice stone size gets larger (*18*). Such difference in the results suggests that pore size also influences the performance of immobilized enzymes and cells (*38*). *P. stuartii* are facultative anaerobes, therefore they require oxygen for their growth. However, oxygen transfer, which supports the growth of immobilized *P. stuartii* cells (*21*), is also affected by the pore size of immobilization support. Pumice stones have irregular pores and varied connectivity (*39*), which might contribute to different results from the previous research.

Fig. 1 also shows that higher pumice stone/growth medium ratio increases the production of *N-*acetylglucosamine. The highest concentration of *N-*acetylglucosamine was obtained from the cells immobilized with pumice stone/growth medium ratio (*m*/*V*) of 1:5. This correlates with the result of the percentage of immobilized cells (Table 1). Higher percentage of immobilized cells means more cells are available to ferment shrimp shell powder, producing higher concentration of *N-*acetylglucosamine. Furthermore, optimum yield can be achieved with the proper carrier amount. Increasing the amount of carrier may provide more space for the free cells to be immobilized, which further leads to higher yield, unless it has reached the optimum value (*36*).

**Fig. 2:**
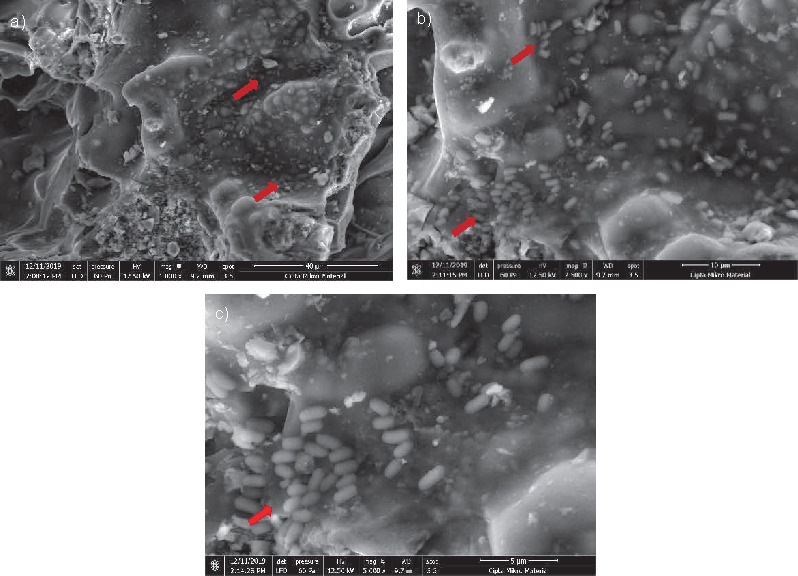
Scanning electron micrographs of *Providencia stuartii* cells immobilized in pumice stone with size of 1.0 cm×1.0 cm×1.0 cm and pumice stone/growth medium ratio of 1:5 observed under the magnification of: a) 1000×, b) 2500×, and c) 5000×. Red arrows show some *P. stuartii* cells that were entrapped in the porous structure of pumice stones

To ensure that *P. stuartii* cells were immobilized in pumice stone, SEM analysis was also done, and the results can be seen in Fig. 2, which shows the scanning electron micrographs of a pumice sample with size of 1.0 cm×1.0 cm×1.0 cm and pumice stone/growth medium ratio of 1:5 after 2 h of *P. stuartii* cell immobilization. The results show the presence of cells that had been immobilized in the pores of the pumice. Hence, these images also prove that pumice stone is suitable for immobilizing *P. stuartii* cells.

**Fig. 3:**
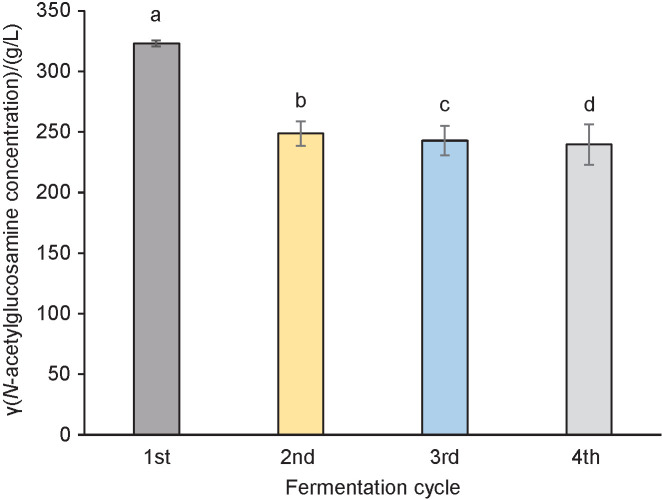
*N-*acetylglucosamine concentration obtained from repeated fermentation cycles. Data are presented as mean value±S.D, *N*=20. Different letters indicate a significant difference (p≤0.05)

After the bacterial cells had been properly immobilized into the pumice stones, repeated fermentation was conducted to determine the stability of the immobilized cells. In this repeated fermentation, pumice stone with the size of 1.0 cm×1.0 cm×1.0 cm and pumice stone/growth medium ratio of 1:5 was used. Statistical result using ANOVA shows that fermentation cycles have a significant effect on the *N-*acetylglucosamine production (p≤0.05). The effect of fermentation cycles on *N-*acetylglucosamine production can be seen in Fig. 3.

Fig. 3 shows that the *N-*acetylglucosamine production significantly decreases from the first to the second cycle. The highest *N-*acetylglucosamine was obtained in the first fermentation cycle, *i.e*. (323.0±2.5) g/L. However, there was no significant difference in the *N-*acetylglucosamine concentration produced from the second to the fourth cycle, with the lowest concentration of (239.6±16.7) g/L.

The decreased concentration of produced acetlyglucosamine in the second cycle of fermentation was also found in a previous research (*40*) using immobilized *Saccharomyces cerevisiae* in rice hulls for ethanol production. This might be caused by leaching of immobilized cells from the pumice stone surface into the fermentation medium due to abrasion effect. This phenomenon leads to a reduction of the number of cells immobilized in the pumice stones, leaving mostly the cells which were entrapped within the pores (*40*). There is a possibility that some of the *N-*acetylglucosamine produced in the first cycle is due to the presence of the free cells leaked from the pumice stones (*19*).

However, the decreasing value was then followed by stable *N-*acetylglucosamine production in the second, third and fourth cycles. The same behaviour was also found in another research with immobilized *Aspergillus niger* mycelium using pumice stones and eight cycles of gluconic acid production (*17*). It was found that after a decrease in the production of gluconic acid from the first to the sixth cycle, the yield in the sixth up to the eighth cycle did not continue to fall. This could be related to the fact that pumice stone has superior mechanical strength to protect the entrapped cells from the shear force (*11*), hence maintaining the number of remaining cells within the pores.

Moreover, the stable production of *N-*acetylglucosamine might also be contributed by the fact that cells within the supporting material had been properly adapted to and sufficiently maintained in the microenvironment of the pumice (*41*). High concentration of *N-*acetylglucosamine production obtained after four cycles of fermentation shows that immobilization technique using pumice stones can be potentially applied for continuous fermentation using *P. stuartii* cells to produce *N-*acetylglucosamine.

## CONCLUSIONS

*N-*acetylglucosamine can be produced through the repeated submerged fermentation from shrimp shell powder. In this research, cells of *Providencia stuartii,* a strong chitinolytic bacteria, were immobilized in pumice stone with the size of 1.0 cm×1.0 cm×1.0 cm and used repeatedly for four cycles of fermentation. The highest concentration of *N-*acetylglucosamine, *i.e.* (331.4±7.3) g/L, was achieved in the first fermentation cycle, which then decreased in the second cycle and remained stable until the fourth cycle of fermentation. These results show the potential of the application of immobilized *P. stuartii* cells in continuous production of *N-*acetylglucosamine from shrimp shells to treat joint damage or osteoarthritis. However, the purity of the obtained *N-*acetylglucosamine should be further analyzed.
